# Predicting Elevated Postvoid Residual Urine Volume Following OnabotulinumtoxinA Treatment for Overactive Bladder: A Pilot Study

**DOI:** 10.1111/luts.70004

**Published:** 2025-01-12

**Authors:** Israel Franco, Marc Schwartz, Kevin Cline, David Glazier, Anand Patel

**Affiliations:** ^1^ Department of Urology Yale School of Medicine New Haven Connecticut USA; ^2^ MS Biostatistics LLC Clermont Florida USA; ^3^ Regional Urology Shreveport Louisiana USA; ^4^ Virginia Urology Richmond Virginia USA; ^5^ Allergan, an AbbVie Company Marlow UK

**Keywords:** botulinum toxins type A, intermittent urethral catheterization, overactive urinary bladder, ROC curve, urinary retention

## Abstract

**Objectives:**

To evaluate possible predictors of elevated postvoid residual volume (PVR) following onabotulinumtoxinA administration in patients with idiopathic overactive bladder (OAB), a condition that may include urinary urgency, frequency, and nocturia, without any identifiable cause or underlying neurological or metabolic condition.

**Methods:**

Adults who had been treated with 100–200 U onabotulinumtoxinA for OAB and had previous failure of other OAB treatments were identified by retrospective review of medical chart data from three urology clinics in the United States treating patients with a variety of urological conditions. A total of 211 patients were allocated to cohorts based on posttreatment PVR < 200 mL (*n* = 173) and ≥ 200 mL (*n* = 38). Logistic regression analyses were performed to evaluate potential predictors of posttreatment PVR ≥ 200 mL, including pretreatment peak urine flow rate (Qmax), average urine flow rate (Qavg), and Modified Liverpool Qmax and Qavg flow index (FI), and to determine whether patient age and baseline PVR were associated with the likelihood of PVR ≥ 200 mL. Patients were excluded if symptoms of OAB were secondary to a neurological condition, they had a PVR > 200 mL within 2 weeks prior to the index therapy or had been treated with other botulinum toxin formulations for a urinary condition.

**Results:**

In the predictor analyses, neither Qmax nor Qavg alone was a likely predictor. Odds ratios of PVR ≥ 200 mL for Modified Liverpool Qmax FI and Qavg were 0.30 (95% CI: 0.08–0.91; *p* = 0.0488) and 0.07 (95% CI 0.01–0.40; *p* = 0.0045), respectively. When patient age and baseline PVR were incorporated into the analyses, results suggested that Qmax, Qavg, Qmax FI, and Qavg FI, as well as increased age and baseline PVR, were likely predictors of elevated posttreatment PVR.

**Conclusions:**

Patients who are older, have high pretreatment PVR values, and have lower pretreatment urine flow indexes and flows may be at increased risk of developing elevated PVR after receiving onabotulinumtoxinA treatment for OAB.

## 1 Introduction

OnabotulinumtoxinA can be used to treat patients with idiopathic overactive bladder (OAB) and urinary incontinence inadequately treated with anticholinergic agents [1, 2, 3]. Some patients who receive onabotulinumtoxinA via intradetrusor injection for OAB experience elevated postvoid residual volume (PVR) that may require clean intermittent catheterization (CIC) [2, 3, 4], which increases the risk of urinary tract infection [4]. The American Urological Association recommends monitoring PVR prior to intradetrusor botulinum toxin injection and 2 weeks after the first botulinum toxin injection [5]. PVR should be performed immediately after voiding, using non‐invasive bladder ultrasound (preferred) or alternatively, straight catheterization.

The definition of an elevated PVR can range from 100 to 400 mL, with limited data available to guide diagnosis and treatment of patients with elevated PVR [6]. Determining which patients are at increased risk for elevated PVR following onabotulinumtoxinA treatment would enhance patient counseling and inform monitoring patients at risk for elevated PVR.

Several patient characteristics have been identified as potential risk factors for adverse outcomes following treatment with onabotulinumtoxinA for OAB. For instance, elevated baseline PVR and voiding efficiency (VE), defined by the formula voided volume (VV)/VV + PVR [7], are significant predictors of adverse events (AEs) among patients treated with onabotulinumtoxinA for OAB [8, 9]. Furthermore, acute urinary retention is more common among patients with baseline VE < 90%, and low baseline VE is a predictor of large PVR at 3 months after onabotulinumtoxinA injection [8, 10]. Increased age has also been associated with elevated PVR among patients with urological symptoms [6]. The current study was performed to determine if it would be possible to predict which patients would be more likely to experience elevated PVR that could result in CIC induction following onabotulinumtoxinA administration using another method of measuring VE (i.e., flow index) derived from free flow rates.

## 2 Methods

This was a retrospective review of medical chart data from three urology clinics in the United States. Electronic medical records were queried using Current Procedural Terminology code 52287 (cystourethroscopy, with injection(s) for chemodenervation of the bladder), then screened to identify male and female patients who had been treated with 100–200 U onabotulinumtoxinA per physicians' usual practice for idiopathic OAB that was inadequately managed with one or more anticholinergic agents [1]. The dosages used were at the discretion of the treating clinician, based on each patients clinical characteristics and treatment needs. Patients were required to be ≥ 18 years of age on the day of their first onabotulinumtoxinA injection and have a negative urine dipstick test for nitrites and leukocyte esterase, or be asymptomatic for urinary tract infection, at that time. Patients were excluded if symptoms of OAB were secondary to a neurological condition, they had a PVR > 200 mL within 2 weeks prior to the index therapy or had been treated with other botulinum toxin formulations for a urinary condition.

Data were collected in accordance with applicable privacy requirements, and no identifying or personal health information was collected without written, informed consent by the patient. During the study, patient information remained confidential and accessible only to investigators and authorized research staff, and all data entry and analyses were conducted using anonymized study identification codes that were destroyed after study completion. De‐identified case report forms were to remain securely stored for a minimum of 2 years or as necessary to conform to local regulations.

For this study, elevated PVR was defined as 200 mL or greater, as this was expected to be high enough to require induction of CIC. During phase 3 studies, symptomatic patients with a PVR of 200 mL or higher may have been catheterized. Only data from patients receiving their first treatment with onabotulinumtoxinA for OAB were used in the analysis. Potential predictors of posttreatment PVR ≥ 200 mL analyzed were VE, pretreatment free flow peak urine flow rate (Qmax), average urine flow rate (Qavg), and flow index (FI) Qmax_actual_/Qmax_estimated_ and Qavg_actual_/Qavg_estimated_, where estimated flows were derived from formulas that predict Qmax and Qavg, created from the previously developed Liverpool nomogram equations [11]. These equations (Equations (1), (2), (3), (4)) were used in the original form and modified to include total bladder capacity (TBC) instead of VV and are referred to in this analysis as the modified Liverpool equations (MLE).

(1) $$\text{Female}\;\mathrm{MLE}\;\text{Qmax}=e^{\left(0.511+0.505\times \ln\left[\mathrm{TBC}\right]\right)}$$

(2) $$\text{Female}\;\mathrm{MLE}\;\text{Qavg}={\left(-0.921+0.869\times \ln\left[\mathrm{TBC}\right]\right)}^{2}$$

(3) $$\text{Male}\;\mathrm{MLE}\;\text{Qmax}={\left(2.37+0.18\times {\mathrm{TBC}}^{0.5}\right)}^{2}$$

(4) $$\text{Male}\;\mathrm{MLE}\;\text{Qavg}={\left(1.8+0.14\times {\mathrm{TBC}}^{0.5}\right)}^{2}$$

As previously demonstrated, the use of the TBC instead of VV is more effective in predicting outcomes than the use of formulas that only measure VV [7, 12, 13]; estimated Qmax and Qavg values by TBC are presented in Table S1. Each potential predictor was analyzed individually, then in combination with patient age and baseline PVR.

### 2.1 Statistical Analysis

No formal sample size determination was performed. Categorical parameters are presented descriptively as number and percentage, and continuous parameters as mean, standard deviation (SD), median, and range.

Logistic regression analyses were used to evaluate whether preselected patient characteristics were associated with the likelihood that PVR would be < 200 versus ≥ 200 mL. Odds ratios (ORs), confidence intervals, and *p* values were derived from the logistic regression models. Receiver operating characteristic (ROC) curves were used to determine whether specific cutoffs (Youden cutoff method [14]) of the potential predictors could be identified that were predictive of PVR. Sensitivity, specificity, accuracy, positive predictive value, and negative predictive value were also determined. Wilcoxon rank sum, two‐sample, nonparametric tests were used to compare the distributions of the quantitative measures (Qmax, Qavg, MLE Qmax FI, and MLE Qavg FI) between the PVR ≥ 200 and < 200 mL cohorts. All statistical analyses were performed using R version 3.5.0 or greater (The R Foundation for Statistical Computing), with the notable exception of the ROC analysis of VE, defined as PVR/(PVR + VV) (shown in Figure S3), which was analyzed using Microsoft Excel with XLStat version 2022.4.1 (Addinsoft, New York, NY).

## 3 Results

### 3.1 Study Population

Posttreatment PVR data were available from 211 patients, who were allocated to posttreatment PVR cohorts of < 200 mL (*n* = 173) and ≥ 200 mL (*n* = 38) (Table 1). Within the elevated PVR cohort, patients had values ranging 200–299 mL (*n* = 25), 300–399 mL (*n* = 7), and ≥ 400 mL (*n* = 6). Demographics and clinical characteristics were well‐balanced between cohorts. Patients in the ≥ 200 mL cohort had failed a mean (± SD) of 2.4 ± 1.4 prior OAB medications and 100% had no prior exposure to botulinum toxins, while patients in the < 200 mL cohort had failed 2.8 ± 1.4 prior OAB medications and 95.4% had no prior botulinum toxin exposure.

**TABLE 1:** Baseline demographics, medical history, and urinary flow parameters.

Characteristic	Posttreatment PVR		*p*
	< 200 mL (*n* = 173)	≥ 200 mL (*n* = 38)	
Age (years)			
Mean ± SD	68.5 ± 13.8	73.3 ± 10.6	—
Female, *n* (%)	140 (80.9)	28 (73.7)	—
Race, *n* (%)			
African American	23 (13.3)	3 (7.9)	—
White	142 (82.1)	33 (86.8)	—
Other/unknown	8 (4.6)	2 (5.3)	—
Botulinum toxin naive, *n* (%)	165 (95.4)	38 (100)	—
Number of failed OAB medications			
Mean ± SD (min, max)	2.8 ± 1.4 (1.0, 8.0)	2.4 ± 1.4 (1.0, 7.0)	—
Baseline PVR (mL)			
Mean ± SD	29.9 ± 50.9	74.8 ± 73.9	—
Qmax			
Mean ± SD	21.3 ± 8.5	24.0 ± 8.7	0.0855
Median (IQR)	20.5 (14.6–27.1)	24.3 (18.0–29.3)	
Qmax FI			
Mean ± SD	0.7 ± 0.4	0.5 ± 0.3	0.0896
Median (IQR)	0.6 (0.4–0.9)	0.5 (0.3–0.8)	
Qavg			
Mean ± SD	11.7 ± 4.8	13.3 ± 4.7	0.0773
Median (IQR)	11.8 (7.9–15.2)	14.2 (10.1–16.8)	
Qavg FI			
Mean ± SD	0.5 ± 0.2	0.3 ± 0.2	0.0020
Median (IQR)	0.4 (0.3–0.6)	0.3 (0.2–0.5)	

Abbreviations: FI, flow index, where flow index = actual flow rate/estimated flow rate; IQR, interquartile range; OAB, overactive bladder; PVR, postvoid residual urine volume; Qavg, average urine flow; Qmax, peak urine flow, SD, standard deviation.

### 3.2 Predictor Analyses

When Qmax and Qavg were each evaluated as individual predictors of elevated posttreatment PVR, trends toward potential relationships with PVR ≥ 200 mL were observed but they were not statistically significant. In the PVR ≥ 200 and < 200 mL cohorts, mean (± SD) Qmax values were 24.0 ± 8.7 and 21.3 ± 8.5 mL/s, respectively (*p* = 0.0855) (Table 1). For the logistic regression analysis, the OR for Qmax versus PVR ≥ 200 mL was 1.04 (95% CI: 1.00–1.08; *p* = 0.0827). In the corresponding ROC analysis, the AUC was 0.5891, sensitivity was 66%, and specificity was 51% (Figure S1). In the Qavg analysis, the mean Qavg was 13.3 ± 4.7 mL/s in the PVR ≥ 200 mL cohort and 11.7 ± 4.8 mL/s in the < 200 mL cohort (*p* = 0.0773) (Table 1). In the logistic regression analysis, the OR for Qavg versus PVR ≥ 200 mL was 1.07 (95% CI: 1.00–1.16; *p* = 0.0727), while the corresponding ROC analysis revealed an AUC of 0.5916, sensitivity of 68%, and specificity of 50% (Figure S1).

Assessment of MLE Qmax FI or MLE Qavg FI as an individual predictor of elevated posttreatment PVR suggested a reduced risk of developing elevated posttreatment PVR with increases in pretreatment MLE Qmax Fl or MLE Qavg Fl. Mean MLE Qmax FI values in the PVR ≥ 200 and < 200 mL cohorts were 0.5 ± 0.3 and 0.7 ± 0.4, respectively (*p* = 0.0896) (Table 1). The logistic regression analysis of MLE Qmax FI versus PVR ≥ 200 mL provided a significant OR of 0.30 (95% CI: 0.08–0.91; *p* = 0.0488). In the corresponding ROC curve, AUC was 0.5880, sensitivity was 95%, and specificity was 23% (Figure S1). In the MLE analysis, the mean Qavg FI in the PVR ≥ 200 mL cohort (0.3 ± 0.2) was significantly increased versus the < 200 mL cohort (0.5 ± 0.2; *p* = 0.0020) (Table 1). The logistic regression analysis of Qavg FI versus PVR ≥ 200 mL was also significant with an OR of 0.07 (95% CI 0.01–0.40; *p* = 0.0045). In the corresponding ROC analysis, the AUC was 0.6603, sensitivity was 71%, and specificity was 61% (Figure S1). Comparison of the original Liverpool equations utilizing VV to the MLE TBC equations indicated good correlation, as low PVR values and greater deviations with increased PVR would be expected (Figure S2). Probability analysis showed that the MLE had better discernibility of elevated postvoid residuals than the original Liverpool formulas, regardless of whether flow rates or flow indexes were being evaluated.

**FIGURE 1:**
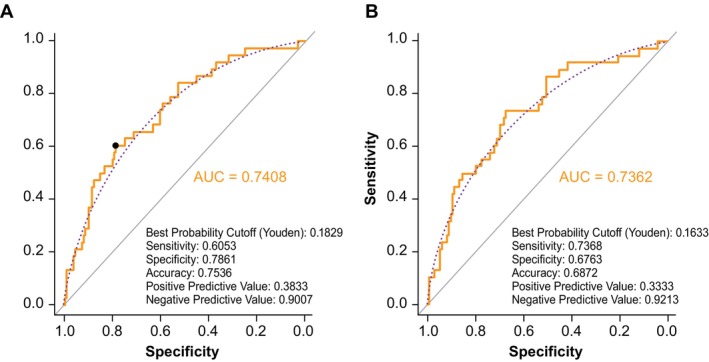
ROC analysis of (a) Qavg and (b) Qavg FI versus PVR ≥ 200 mL with the addition of age and baseline PVR volume. AUC, area under the curve; FI, flow index; PVR, postvoid residual urine volume; Qavg, average urine flow; ROC, receiver operating characteristic.

When age was added to Qmax, the OR versus PVR ≥ 200 mL in the logistic regression analysis was 1.03 (95% CI: 1.00–1.07; *p* = 0.0601), while adding baseline PVR to Qmax resulted in an OR of 1.01 (95% CI: 1.00–1.02; *p* = 0.0025) (Table 2). In the ROC analysis of Qmax with age and baseline PVR, the AUC was 0.7378, sensitivity was 61%, and specificity was 77%. When patient age was added to Qavg, the OR versus ≥ 200 mL PVR was 1.03 (95% CI: 1.00–1.07; *p* = 0.0570), while the addition of baseline PVR to Qavg resulted in an OR of 1.01 (95% CI: 1.00–1.02; *p* = 0.0028) (Table 2). In the ROC analysis of Qavg with age and baseline PVR, the AUC was 0.7408, sensitivity was 60%, and specificity was 79% (Figure 1A).

**TABLE 2:** Logistic regression analysis of Qmax, Qavg, Liverpool Qmax FI, and Qavg FI versus PVR ≥ 200 mL with the addition of age and baseline PVR as covariates.

Model	Estimate ± SE	Odds ratio (95% CI) of PVR ≥ 200 mL	*p*
Qmax			
Qmax	−0.0150 ± 0.0277	0.99 (0.93–1.04)	0.5885
Age, years	0.0320 ± 0.0170	1.03 (1.00–1.07)	0.0601
Baseline PVR (mL)	0.0108 ± 0.0036	1.01 (1.00–1.02)	0.0024
Qmax FI			
Qmax FI	−0.0915 ± 0.6316	0.91 (0.24–2.91)	0.8849
Age, years	0.0319 ± 0.0176	1.03 (1.00–1.07)	0.0690
Baseline PVR (mL)	0.0094 ± 0.0029	1.00 (1.00–1.02)	0.0014
Qavg			
Qavg	−0.0186 ± 0.0491	0.98 (0.89–1.08)	0.7046
Age, years	0.0324 ± 0.0170	1.03 (1.00–1.07)	0.0570
Baseline PVR (mL)	0.0104 ± 0.0035	1.01 (1.00–1.02)	0.0028
Qavg FI			
Qavg FI	−1.1073 ± 0.9916	0.33 (0.04–2.12)	0.2642
Age, years	0.0281 ± 0.0172	1.03 (1.00–1.07)	0.1031
Baseline PVR (mL)	0.0081 ± 0.0030	1.01 (1.00–1.01)	0.0062

Abbreviations: CI, confidence interval; FI, flow index; IQR, interquartile range; PVR, postvoid residual urine volume; Qavg, average urine flow; Qmax, peak urine flow; SE, standard error.

**FIGURE 2:**
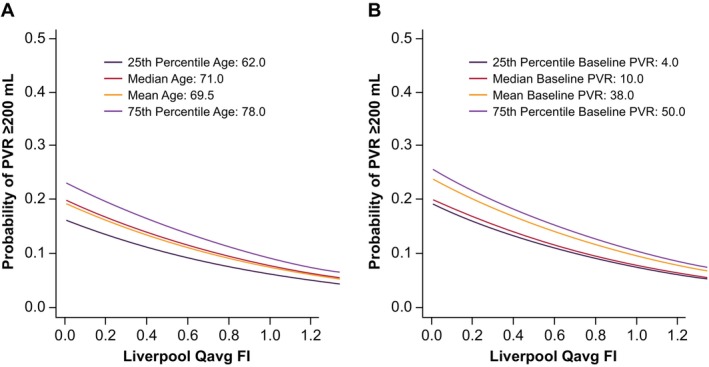
Probability of PVR ≥ 200 mL versus Liverpool Qavg FI with (a) patient age set to the median and (b) baseline PVR set to the median. FI, flow index; PVR, postvoid residual urine volume; Qavg, average urine flow.

Similar combined analyses were performed for MLE Qmax FI and Qavg FI. For MLE Qmax FI, adding patient age resulted in an OR versus ≥ 200 mL PVR of 1.03 (95% CI:1.00–1.07; *p* = 0.0690), while adding baseline PVR to MLE Qmax FI resulted in an OR of 1.00 (95% CI: 1.00–1.02; *p* = 0.0014). Similar results were observed with MLE Qavg FI, as adding patient age resulted in an OR of 1.03 (95% CI: 1.00–1.07; *p* = 0.1031) while adding baseline PVR resulted in an OR of 1.01 (95% CI: 1.00–1.01; *p* = 0.0062) (Table 2). In the ROC analysis, the AUC was 0.7362, sensitivity was 74%, and specificity was 68% (Figure 1B). As shown in Figure 2, the probability of achieving posttreatment PVR > 200 mL decreases as MLE Qavg FI increases for all patients, but the overall risk increases with increased age and baseline PVR. In the ROC analysis of VE (*n* = 211), defined as PVR/(PVR + VV), the AUC was 0.716, the sensitivity was 0.684, and the specificity was 0.665 (Figure S3).

## 4 Discussion

Results of this exploratory analysis suggest that older patients with high pretreatment PVR values and a lower pretreatment urine flow may be at higher risk of developing an elevated PVR following onabotulinumtoxinA treatment for OAB. In addition, a flow index that utilizes TBC in estimated flow calculations, whether maximum or average, improved predictive capabilities vs. VV formulas or Qmax and Qavg. Similar findings were observed in previous studies that applied flow indexes utilizing TBC as the independent variable to better predict outcomes based on flow rates [7, 12, 13]. Because flow index normalizes differences that may be due to patients' sex, it is an appropriate measure of voiding efficiency [7, 13, 15].

These results are important, as predicting which patients may have an increased risk of elevated posttreatment PVR following onabotulinumtoxinA treatment, and thus may require CIC, would allow more effective, individualized counseling for patients, and for more specific monitoring for those patients at risk. In phase 3 clinical trials evaluating onabotulinumtoxinA for the treatment of patients with OAB, the proportion of patients who developed a PVR ≥ 200 mL was low (8.7% to 8.8%) [2, 3]. Notably, in this analysis, only 13 of 211 patients (6%) developed a PVR > 300 mL after onabotulinumtoxinA administration. In addition, it is thought that many patients who are at low risk of elevated PVR and subsequent CIC do not utilize onabotulinumtoxinA because they mistakenly believe that their risk of these adverse effects is high. By providing accurate risk stratification, better counseling will allow patients who may have otherwise incorrectly perceived high risk associated with onabotulinumtoxinA use to benefit from onabotulinumtoxinA treatment. Likewise, this will allow an informed decision regarding the benefits and risks of onabotulinumtoxinA treatment for patients at high risk of elevated PVR.

These data can be used to develop preliminary nomograms or formulas to help physicians better quantify the risk of elevated PVR for individual patients treated with onabotulinumtoxinA to assist with counseling and follow‐up monitoring, similar to those previously generated for other urinary conditions [16, 17, 18]. As the nomograms are refined with more data, these preliminary nomograms can guide urologists' clinical decision‐making when considering which patients are candidates for injection. For example, from our current analysis, approximately 600 patients would be needed to develop a nomogram, assuming three continuous variables with no other transformation of continuous variables and a conservative prevalence of PVR at the 200 mL threshold of 10%. Further studies are needed to produce additional nomograms based on these data that can be used by urologists in clinical practice.

This study was limited by the retrospective design and limited number of patients with elevated PVR values. The results are preliminary and should be interpreted with caution, particularly in light of the small sample size of the PVR > 200 mL group. In addition, the analysis did not control for potential interdependence among the factors that were examined as predictors of elevated PVR. Due to the small sample size and exploratory nature of this analysis, we are unable to reliably predict a risk cutoff for all three continuous variables simultaneously (age, Qmax, and PVR). Follow‐up analyses should be performed in larger patient populations and employ continuous statistical methodology to confirm and extend the findings from this exploratory analysis.

## 5 Conclusion

The results of this study suggest that patients who are older and have high pretreatment PVR or lower pretreatment urine flow may be at higher risk of developing an elevated PVR following onabotulinumtoxinA, increasing the risk of CIC induction. Few patients develop a PVR > 200 mL and even fewer develop a PVR > 300 mL, so retention concerns should be placed in context of risk. These preliminary results may aid clinicians in identifying which patients may require closer monitoring after receiving onabotulinumtoxinA and allow an informed decision weighing the risks and benefits of onabotulinumtoxinA treatment.

## Author Contributions

**Israel Franco:** conceptualization, data curation, formal analysis, methodology, writing – review and editing. **Marc Schwartz:** formal analysis, writing – review and editing. **Kevin Cline:** investigation, writing – review and editing. **David Glazier:** investigation, writing – review and editing. **Anand Patel:** conceptualization, data curation, methodology, writing – review and editing.

## Ethics Statement

Ethics approval was obtained from Advarra Inc. (formerly Chesapeake IRB), located in Columbia, MD, USA.

## Consent

Data were collected in accordance with applicable privacy requirements, and no identifying or personal health information was collected from a patient without written, informed consent.

## Conflicts of Interest

Israel Franco is a consultant to Allergan, an AbbVie Company. Marc Schwartz is a paid biostatistical consultant to Allergan, an AbbVie Company. Kevin Cline and David Glazier declare no conflicts of interest. Anand Patel and Anita Preininger are an employee of AbbVie and may hold AbbVie stock.

## Supporting information


Data S1.

## Data Availability

AbbVie is committed to responsible data sharing regarding the clinical trials we sponsor. This includes access to anonymized, individual, and trial‐level data (analysis data sets), as well as other information (e.g., protocols, clinical study reports, or analysis plans), as long as the trials are not part of an ongoing or planned regulatory submission. This includes requests for clinical trial data for unlicensed products and indications. These clinical trial data can be requested by any qualified researchers who engage in rigorous, independent, scientific research, and will be provided following review and approval of a research proposal, Statistical Analysis Plan (SAP), and execution of a Data Sharing Agreement (DSA). Data requests can be submitted at any time after approval in the United States and Europe and after acceptance of this manuscript for publication. The data will be accessible for 12 months, with possible extensions considered. For more information on the process or to submit a request, visit the following link: https://vivli.org/ourmember/abbvie/ then select “Home.”
