# Supplementary material for: Predicting Elevated Postvoid Residual Urine Volume Following OnabotulinumtoxinA Treatment for Overactive Bladder: A Pilot Study
Source: Low Urin Tract Symptoms. 2025 Jan 12;17(1):e70004. doi: 10.1111/luts.70004 (PMC11725389; doi:10.1111/luts.70004)
Supplement: Supplementary file 1 — Data S1. [file LUTS-17-e70004-s001.docx]

# Supplemental Material

**Supplemental Table S1.** Estimated Modified Liverpool Qmax and Qavg for Females and Males by TBC

| **TBC** | **Female Modified Liverpool Qmax** | **Female Modified Liverpool Qavg** | **Male Modified Liverpool Qmax** | **Male Modified Liverpool Qavg** |
| --- | --- | --- | --- | --- |
| 100 | 17.1 | 9.5 | 17.4 | 10.2 |
| 110 | 17.9 | 10.0 | 18.1 | 10.7 |
| 120 | 18.7 | 10.5 | 18.9 | 11.1 |
| 130 | 19.5 | 10.9 | 19.6 | 11.5 |
| 140 | 20.2 | 11.4 | 20.2 | 11.9 |
| 150 | 20.9 | 11.8 | 20.9 | 12.4 |
| 160 | 21.6 | 12.2 | 21.6 | 12.8 |
| 170 | 22.3 | 12.5 | 22.2 | 13.1 |
| 180 | 23.0 | 12.9 | 22.9 | 13.5 |
| 190 | 23.6 | 13.2 | 23.5 | 13.9 |
| 200 | 24.2 | 13.6 | 24.2 | 14.3 |
| 210 | 24.8 | 13.9 | 24.8 | 14.7 |
| 220 | 25.4 | 14.2 | 25.4 | 15.0 |
| 230 | 26.0 | 14.5 | 26.0 | 15.4 |
| 240 | 26.5 | 14.8 | 26.6 | 15.8 |
| 250 | 27.1 | 15.0 | 27.2 | 16.1 |
| 260 | 27.6 | 15.3 | 27.8 | 16.5 |
| 270 | 28.2 | 15.6 | 28.4 | 16.8 |
| 280 | 28.7 | 15.8 | 29.0 | 17.2 |
| 290 | 29.2 | 16.0 | 29.5 | 17.5 |
| 300 | 29.7 | 16.3 | 30.1 | 17.8 |
| 310 | 30.2 | 16.5 | 30.7 | 18.2 |
| 320 | 30.7 | 16.7 | 31.2 | 18.5 |
| 330 | 31.2 | 17.0 | 31.8 | 18.9 |
| 340 | 31.6 | 17.2 | 32.4 | 19.2 |
| 350 | 32.1 | 17.4 | 32.9 | 19.5 |
| 360 | 32.6 | 17.6 | 33.5 | 19.9 |
| 370 | 33.0 | 17.8 | 34.0 | 20.2 |
| 380 | 33.5 | 18.0 | 34.6 | 20.5 |
| 390 | 33.9 | 18.2 | 35.1 | 20.8 |
| 400 | 34.4 | 18.4 | 35.6 | 21.2 |
| 410 | 34.8 | 18.6 | 36.2 | 21.5 |
| 420 | 35.2 | 18.7 | 36.7 | 21.8 |
| 430 | 35.6 | 18.9 | 37.2 | 22.1 |
| 440 | 36.0 | 19.1 | 37.8 | 22.4 |
| 450 | 36.5 | 19.3 | 38.3 | 22.8 |
| 460 | 36.9 | 19.4 | 38.8 | 23.1 |
| 470 | 37.3 | 19.6 | 39.3 | 23.4 |
| 480 | 37.7 | 19.7 | 39.9 | 23.7 |
| 490 | 38.1 | 19.9 | 40.4 | 24.0 |
| 500 | 38.5 | 20.1 | 40.9 | 24.3 |
| 510 | 38.8 | 20.2 | 41.4 | 24.6 |
| 520 | 39.2 | 20.4 | 41.9 | 24.9 |
| 530 | 39.6 | 20.5 | 42.4 | 25.2 |
| 540 | 40.0 | 20.7 | 42.9 | 25.5 |
| 550 | 40.3 | 20.8 | 43.4 | 25.8 |
| 560 | 40.7 | 21.0 | 44.0 | 26.1 |
| 570 | 41.1 | 21.1 | 44.5 | 26.4 |
| 580 | 41.4 | 21.2 | 45.0 | 26.7 |
| 590 | 41.8 | 21.4 | 45.5 | 27.0 |
| 600 | 42.2 | 21.5 | 46.0 | 27.3 |
| 610 | 42.5 | 21.6 | 46.5 | 27.6 |
| 620 | 42.9 | 21.8 | 46.9 | 27.9 |
| 630 | 43.2 | 21.9 | 47.4 | 28.2 |
| 640 | 43.6 | 22.0 | 47.9 | 28.5 |
| 650 | 43.9 | 22.2 | 48.4 | 28.8 |
| 660 | 44.2 | 22.3 | 48.9 | 29.1 |
| 670 | 44.6 | 22.4 | 49.4 | 29.4 |
| 680 | 44.9 | 22.5 | 49.9 | 29.7 |
| 690 | 45.2 | 22.7 | 50.4 | 30.0 |
| 700 | 45.6 | 22.8 | 50.9 | 30.3 |
| 710 | 45.9 | 22.9 | 51.4 | 30.6 |
| 720 | 46.2 | 23.0 | 51.8 | 30.9 |
| 730 | 46.5 | 23.1 | 52.3 | 31.2 |
| 740 | 46.9 | 23.2 | 52.8 | 31.5 |
| 750 | 47.2 | 23.3 | 53.3 | 31.7 |
| 760 | 47.5 | 23.5 | 53.8 | 32.0 |
| 770 | 47.8 | 23.6 | 54.2 | 32.3 |
| 780 | 48.1 | 23.7 | 54.7 | 32.6 |
| 790 | 48.4 | 23.8 | 55.2 | 32.9 |
| 800 | 48.8 | 23.9 | 55.7 | 33.2 |
| 810 | 49.1 | 24.0 | 56.1 | 33.5 |
| 820 | 49.4 | 24.1 | 56.6 | 33.7 |
| 830 | 49.7 | 24.2 | 57.1 | 34.0 |
| 840 | 50.0 | 24.3 | 57.6 | 34.3 |
| 850 | 50.3 | 24.4 | 58.0 | 34.6 |
| 860 | 50.6 | 24.5 | 58.5 | 34.9 |
| 870 | 50.9 | 24.6 | 59.0 | 35.2 |
| 880 | 51.2 | 24.7 | 59.4 | 35.4 |
| 890 | 51.4 | 24.8 | 59.9 | 35.7 |
| 900 | 51.7 | 24.9 | 60.4 | 36.0 |
| 910 | 52.0 | 25.0 | 60.8 | 36.3 |

Qavg, average urine ﬂow; Qmax, peak urine ﬂow; TBC, total bladder capacity.

**Supplemental Figure S1.** ROC analyses of (a) Qmax, (b) Liverpool Qmax FI, (c) Qavg, and (d) Liverpool Qavg FI vs PVR ≥200 mL


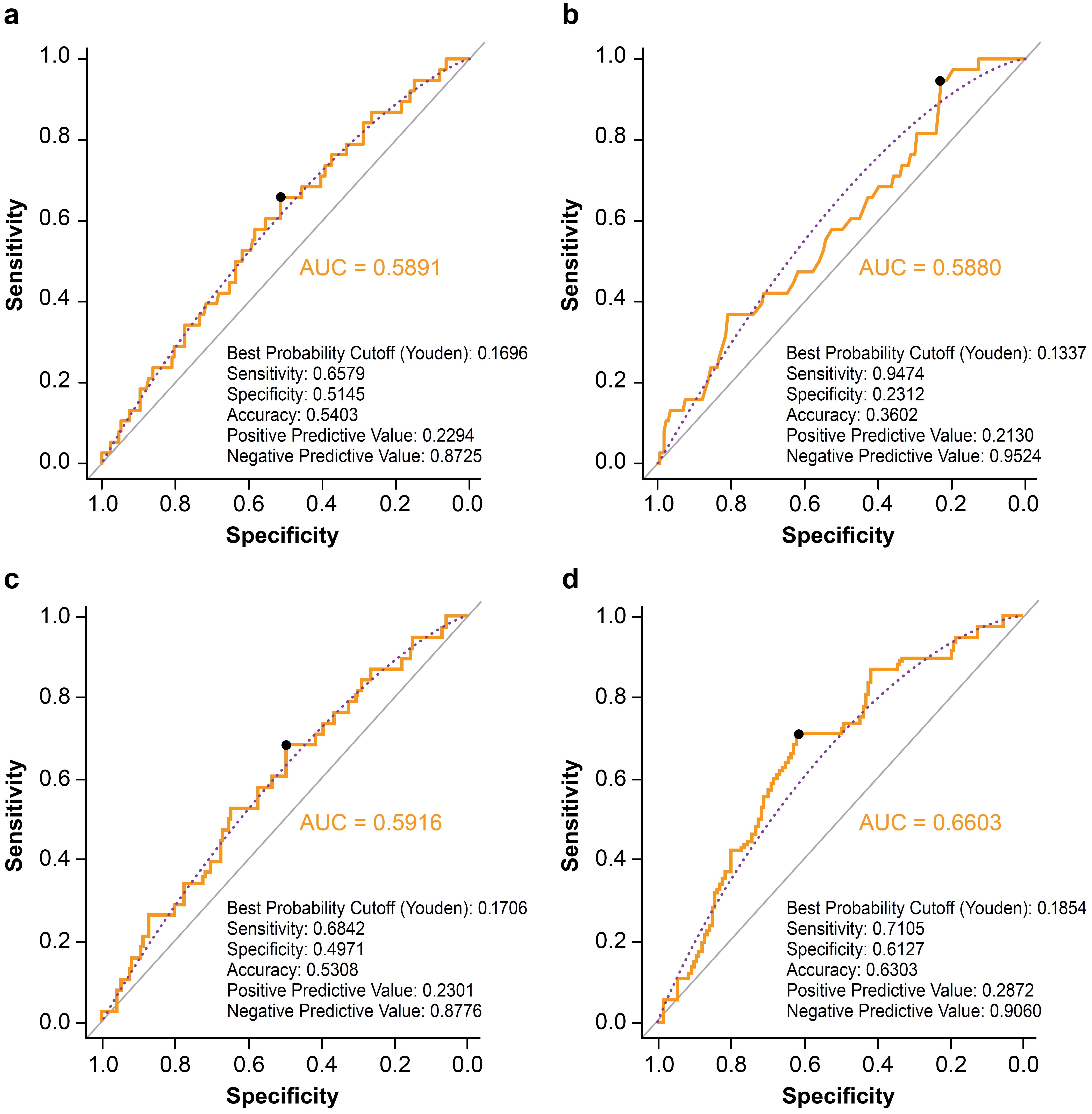


AUC, area under the curve; FI, flow index; PVR, postvoid residual urine volume; Qavg, average urine flow; Qmax, pretreatment peak urine flow; ROC, receiver operating characteristic.

**Supplemental Figure S2.** Pairs scatterplot of selected covariate measures – Spearman scatterplots


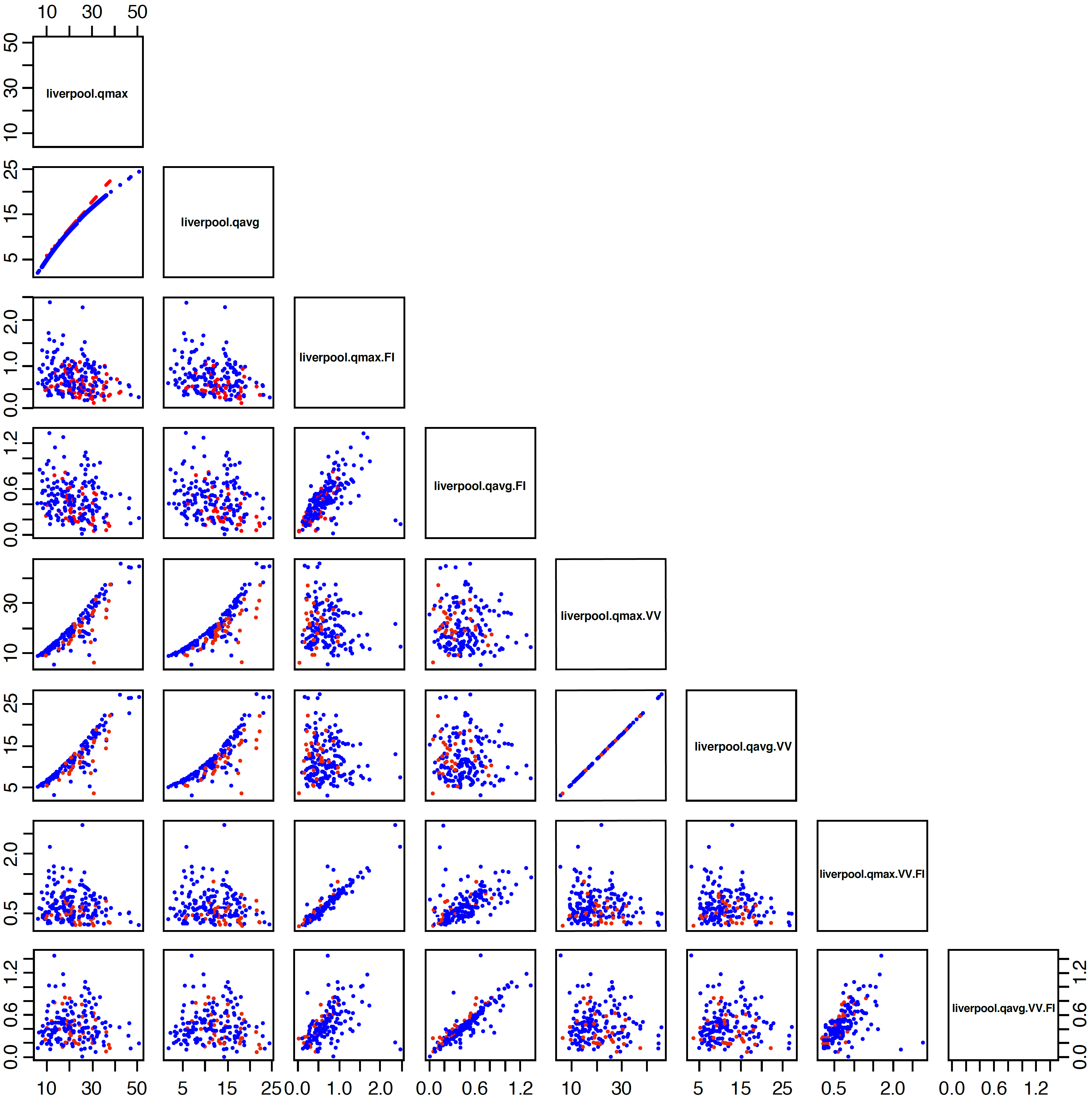


Red = Male; Blue = Female.

**Supplemental Figure S3.** ROC analysis of VE vs PVR >200 mL


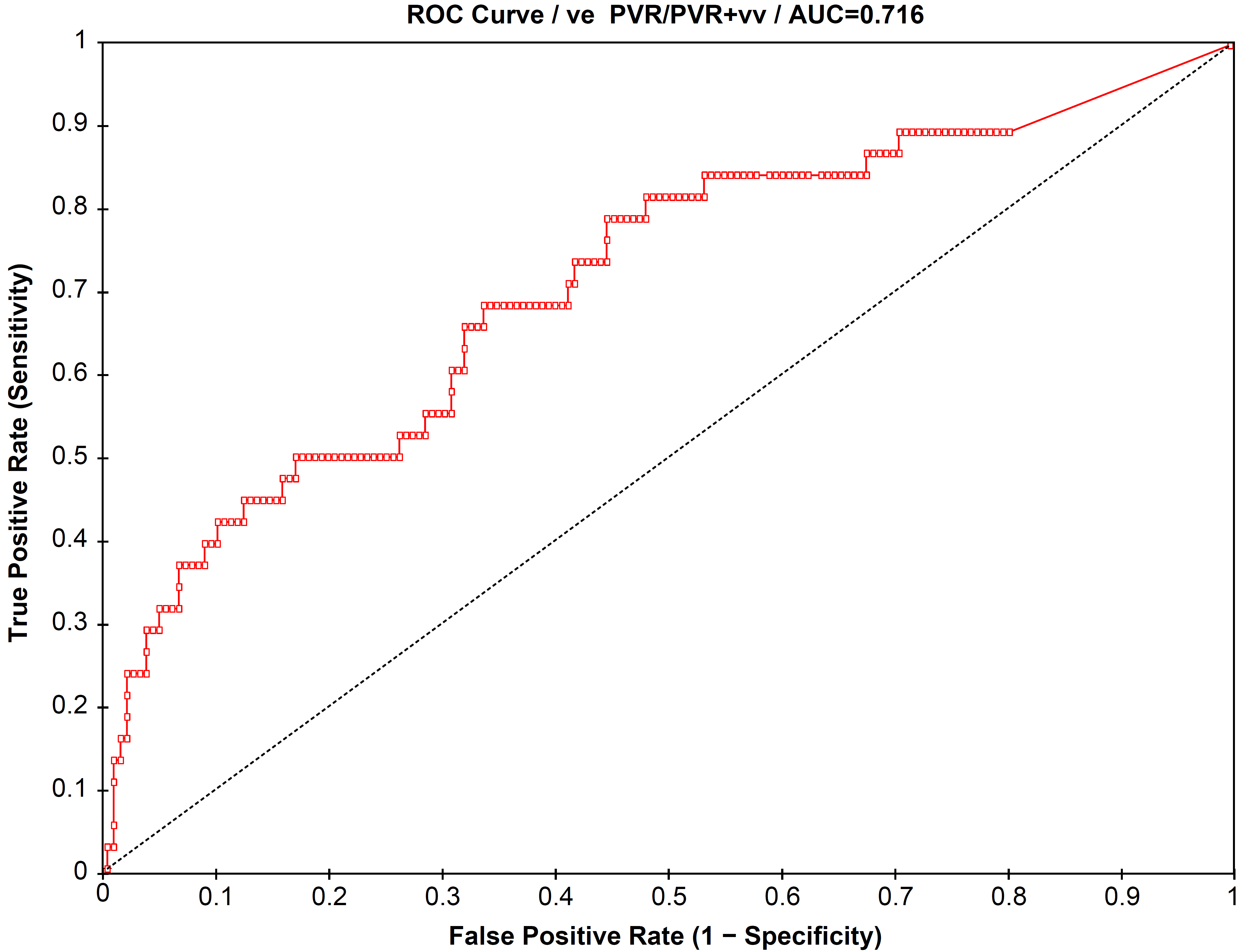


AUC, area under the curve; PVR, postvoid residual urine volume; ROC, receiver operating characteristic; VE, voiding efficiency; VV, voided volume.
